# X-Ray Contrast Media Mechanisms in the Release of Mast Cell Contents: Understanding These Leads to a Treatment for Allergies

**DOI:** 10.1155/2011/276258

**Published:** 2011-09-15

**Authors:** Elliott C. Lasser

**Affiliations:** UCSD Emeritus, USA

## Abstract

A long history of searching for the etiology of X-ray contrast material (CM) reactions has led to the understanding that the CM do not produce anti-CM antigens. Since CM reactions are anaphylactoid in nature, however, a source for mast cell activation was sought. This resulted in the finding that concentrated CM could suppress mast cell activation by attachment to the Fc portion of IgE and IgG. This is presumed to be a steric hindrance effect. In a study of the effects of CM on BP and a study of the effects of CM in sensitized rats, it was concluded that less concentrated CM activated mast cells and that this mechanism was best explained by bridging of adjacent IgE molecules via attachment to their Fc segments. The mast cell release of heparin activating the contact system, as well as the release of histamine, is believed to be responsible for CM reactions and allergic diatheses.

## 1. Contrast Media Molecular Structures

**Figure 1:**
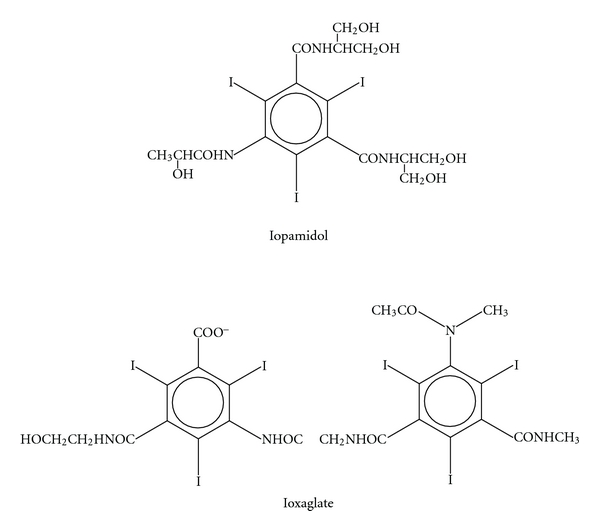
Representative current X-ray contrast molecules. Iopamidol is a nonionic monomer. Ioxaglate is an ionic dimer. All current contrast media have iodide atoms on the 2, 4, and 6 positions on the benzene ring.

Currently all X-ray contrast media (CM) for intravascular opacification are tri-iodinated benzene moieties that are fully substituted and occur in either a monomer or a dimer form (Figure 1).

## 2. The Contact System in CM Anaphylaxis

**Figure 2:**
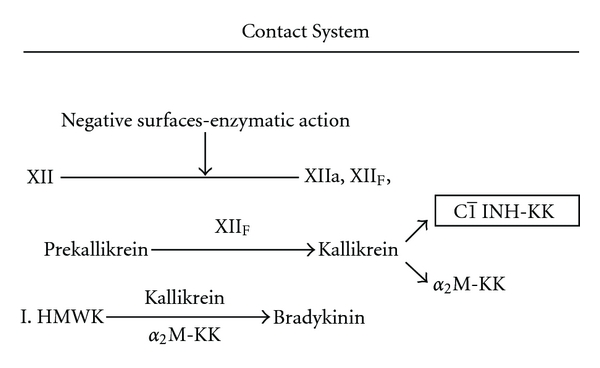
The 3 major proteins of the contact system Factor XII, Prekallikrein, and high molecular weight kininogen are depicted. The major role played by the C1 inhibitor is noted. Factor XIIf is a breakdown product of Factor XII.

The contact system is made up of three proteins (Figure 2): (1) Factor XII, the protein that initiates activation of the intrinsic coagulation system and can be activated by negative surfaces in the circulation, (2) prekallikrein, and (3) high molecular weight kininogen. Activation of Factor XII converts prekallikrein to kallikrein, which converts high molecular kininogen to bradykinin. Bradykinin has essentially the same physiologic effects as histamine but is significantly more effective on a mole per mole basis. Furthermore, bradykinin, once formed, can metabolize arachidonic acid into vasoactive prostaglandins and leukotrienes, all of which are known to participate in anaphylactic events.

## 3. Activation of the Contact System and Negative Surfaces

**Figure 3:**
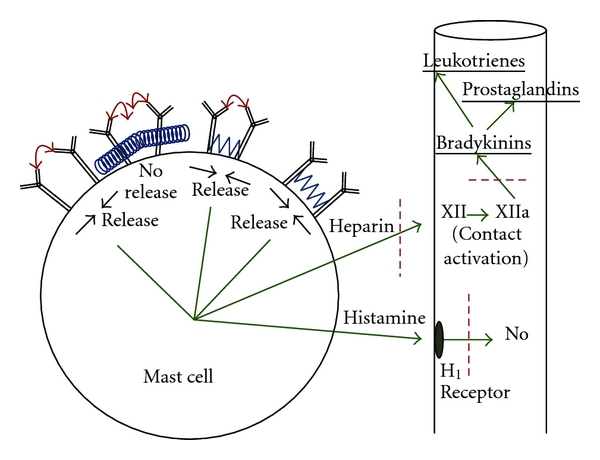
The mast cell dynamics are depicted. Mast cell release occurs when the IgE receptors aggregate and come closer to one another. IgE on the cell is shown to be bridged by antigens (red arrows) binding to the Fab, segment, resulting in mast cell release. Release also occurs in the presence of antigen attachment plus low concentration CM attachment (blue wave forms). Finally, release occurs when there is low CM concentration attachment alone. High concentrations of CM are thought to act by steric hindrance alone and/or by sterically dislodging bound true antigens. The red dashes represent points of possible corticosteroid inhibitory effect.

The elements mentioned above constitute in outline what is known about some of the important mechanisms that play a role in anaphylaxis and allergy. Now, it is necessary to understand what leads up to these events and what can be done to limit them. Negative surfaces in the circulation can activate the contact system. In the body negative surfaces exist in a number of forms. The activation of factor XII will occur when these surfaces have access to this factor in sufficient concentration. In vitro, we have shown that high molecular weight dextran sulfate can activate the system when the temperature is sufficiently low to inhibit plasma substances that would otherwise impede this activation [1]. More importantly, we have demonstrated that this process proceeds more rapidly in atopic or asthmatic individuals than in individuals without these abnormalities [1]. Asthmatics can be demonstrated to have higher concentrations than do nonasthmatics of heparin/heparan sulfate when these molecules are isolated from their neutralizing substances [2, 3] (Figure 3).

## 4. The Role of Heparin-Like Substances

**Figure 4:**
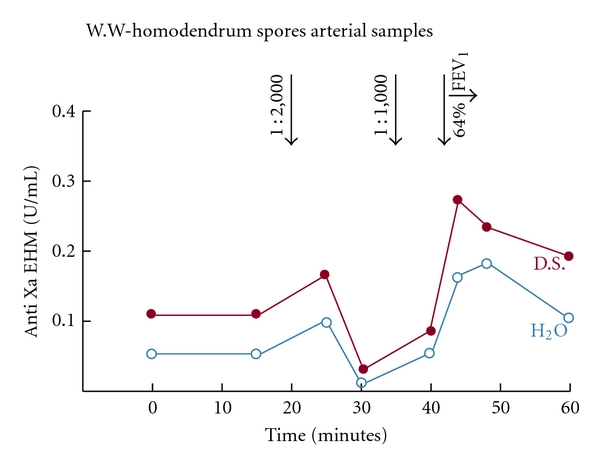
In this study, 6 patients were subjected to inhalational challenge with antigens to which they were known to be sensitive [5]. Simultaneous samples of blood were drawn from the brachial artery and from the antecubital vein. A representative patient is shown here. EHM is endogenous heparin-like material, measured with a functional anti-Xa assay after removing neutralizing substances from the heparin by adding low molecular weight dextran sulfate. After the 1 : 1000 spore challenge, there is an immediate rise in both the EHM material produced by exposing plasma to low molecular weight dextran (red line) and the material with heparin neutralizing substances intact when water, rather than dextran sulfate, was added to the plasma (blue line). A concurrent fall in FEV1 occurs along with the rise in heparin levels when the 1 : 1,000 antigen was inhaled. Similar findings occurred in 3 of the 6 patients.

What are the sources of these heparin-like molecules? While heparan sulfate can be found in a number of tissues, including endothelial linings, the most obvious source of acute supply of heparin-like molecules is the mast cell (Figure 4). When activated, these cells discharge a large number of substances, including heparin, histamine, tryptase, chymase, and ACE and a number of cytokines and chemokines. The in vivo activation of the mast cells, in turn, results when IgE attached to specific receptors on the mast cell surfaces combine with specific antigens in the circulation to produce aggregation of adjacent receptors [4]. (In all our references to the mast cell in this paper, it is understood that basophils in the circulation largely exhibit similar characteristics.) How does heparin/heparan become the negative surface that will activate the contact system in CM reactions? The answer to this lies in putting together two counterintuitive investigations carried out approximately 27 years apart.

## 5. The Attachment of CM to IgE and IgG (The Fallacy of Anticipated Data)

In the first of the counterintuitive experiments mentioned above, dogs underwent 27 injections of iothalamate (Conray 60, Mallinckrodt) into the pulmonary artery and blood samples were collected from a catheter in the left ventricle. The iothalamate was injected in the same volume (30 mL) in each animal but at different injection rates (2 mL/sec and 39 mL/sec). The left ventricular samples were assayed for total histamine content over a 5-minute interval. To our surprise, the maximal histamine blood levels were higher in 20 of the 27 injections when the CM was injected more slowly [6]. In the second of the two experiments, RBC hemagglutination inhibition studies were carried out with several stereotypical CM molecules. The CM used were the same concentrations carried in the commercial vials. (141–320 mgI/mL). In the study, several different antigens were used. While all the tested CM interfered with the antibody-antigen reactivity at different concentrations, the ionic and nonionic dimers and nonionic monomers accomplished this at lesser concentrations of CM than did the ionic monomers [7] (Figure 1). This was counterintuitive, since the interference implied that the CM attached to the antibodies to the exclusion of the specific antigens to these antibodies. Attachment to the antibody by the CM was reasoned to be at the basis of IgE activation of mast cells as was the case with all antigens. In the case of true antigens, the best attachment to the immunoglobulin (at Fab) produces the most activation of the mast cell [7]. In the hemagglutination inhibition study, however, the best attachment was to the CM most unlikely to clinically activate the IgE on mast cells. Then, remembering the puzzling histamine release results in the study done 27 years earlier, a realization dawned on us. In the earlier experiment, we were looking at a SUPPRESSION of IgE activated mast cell activity by the faster injection, not the expected ACTIVATION! The target tissue in this experiment was the lung, and the histamine release from pulmonary mast cells was detected in the heart so that the release was modulated by a high concentration of CM not usually available in other peripheral target tissues. 

We also realized that the CM must attach to the Fc (constant) portion of the immunoglobulins and not the Fab (variable) portion where most antigens attached, since the attachment occurred with multiple antigens and was therefore nonspecific. In a limited subsection of this study, we found that CM could apparently detach specific antigens if the antigen-antibody complex were shaken for a few minutes before the CM was added.

## 6. CM Attachment to the Immunoglobulins

A number of studies to substantiate the foregoing were then carried out. The first was that the CM, attaching to the constant portion of the immunoglobulin, then had the potential to interfere with attachment of specific antigens to the variable part of the immunoglobulin. This was borne out in follow-up studies [8]. Further, we must come to terms with the fact that in vivo and in vitro, CM, in fact, could activate as well as suppress mast cell activity. To accommodate this fact, it was necessary to postulate that at lesser concentrations attachment of CM to Fc portions of immunoglobulins may activate IgE and lead to the adverse reactions sometimes associated with intravascular CM injections, while at higher concentrations, CM will inhibit activation (Figure 3). On a theoretical basis, this now seemed reasonable, since all the CM are known to show some degree of aggregation at higher concentrations (all the CM have a calculated osmolality that is higher than their experimental values) [9]. It seems probable that at the higher concentrations the CM aggregation produces a steric hindrance effect that limits the necessary receptor aggregation for mast cell activation. The other finding that should be emphasized is that CM are completely nonspecific; that is, they can interfere with any antigen binding to IgG or IgE, and thus merit consideration in any circumstance where CM are present and antigen-antibody reactions take place.

## 7. Where and Why Do the CM Bind to IgE?

In our studies that showed that all of the CM could attach to the Fc portion of immunoglobulins and that the ionic and nonionic dimers bound at lesser concentrations than the monomers, it must be assumed that it is the iodine atoms on the benzene rings that are mainly responsible for the binding to the Fc segments (Figures 1 and 3). This assumption is justified, since the iodines are the only commonality amongst the various CM, there are more iodines available on the dimer than on the monomer, and less molar concentration of the dimer is necessary to inhibit antigen attachment on the RBC hemagglutination assay. Finally, it should be noted that iodine has been shown to bind to some carbohydrate structures although this may be specific for amylose and elementary iodine [10]. Central carbohydrates are present on the Fc structure of these immunoglobulins, and while we have no proof, these would seem to be the most obvious site for these bindings.

## 8. Further Data in Proof

**Figure 5:**
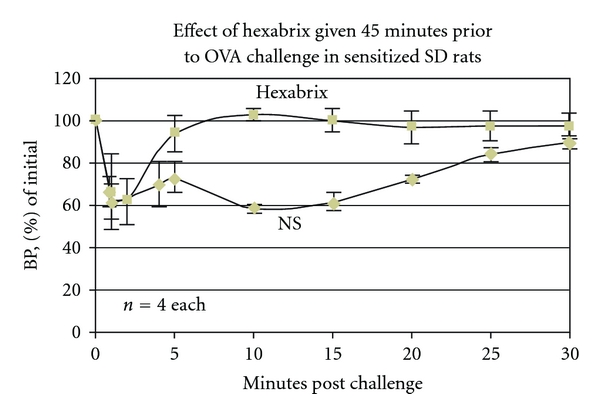
Rats sensitized to ovalbumin and then challenged with ovalbumin, displayed significantly different BP patterns in the presence of either Hexabrix or normal saline. With Hexabrix, whether given 45 minutes earlier, at the time of antigen challenge, or at the nadir of BP levels, there was an abrupt upward swing in BP levels in contradistinction to the prolonged fall in the presence of the saline.

To substantiate the considerations put forward in the above paragraphs, a number of studies were done: PCA (passive cutaneous anaphylaxis) rat study done with an ionic monomer, meglumine/sodium diatrizoate (Angiovist 370, Bracco Diagnostics), showed an inhibition of immune mediated permeability in rough proportion to the CM used [8]. In a study of BP changes in rats injected IV with various CM, the CM demonstrating the poorest (least) binding to antibodies in the RBC hemagglutination inhibition tests (the ionic monomers) showed a drop in BP on injection proportionate to the volumes injected [8]. The CM with better binding (the ionic and nonionic dimers) showed an increase in B.P. on injection, again in proportion to the volumes injected [8]. Injections of 2 strains of rats with diphenhydramine, (Benadryl, Mc Neil-PPC) at a dose of 5 mg/kg also produced a rise in BP as did injections of L-Name, an inhibitor of nitric oxide [8]. Sensitized rats challenged with the sensitizing antigen and then injected with either a dimer CM or saline showed that the saline injection continued the fall in BP occasioned by the challenge, while the CM produced an upward change in the BP [8]. (Figure 5). These studies were interpreted to show that the CM with better (increased) binding to the Fc portions of IgE inhibited mast cell activity, and thereby diminished the resultant basal blood histamine and NO levels producing higher BP.

## 9. Test of the Concepts in Humans

To test our assumptions that the construct that we had put together would have a role in human allergy, we arranged for a 20-person study of allergic rhinitis with ioxaglate 320 (Hexabrix, Guerbet) versus placebo, in individuals with tested sensitivities to the applied antigens [11]. This was a double-blinded crossover study, and the antigen was applied on pads to the nasal mucosa 20 minutes after 2 drops of ioxaglate 320 or saline were dropped into the nostril. In these studies, the patients score the usual symptoms of nasal allergy on a 1+ to 3+ scale. Without going into details, the application of the CM produced fewer symptoms than the placebo in all evaluations. Significant differences (*t*-test) were found for “sneezing” (*P* = 0.018) and “runny nose” (*P* = 0.048) and the combination of “sneezing”, “runny nose” and “itching” was (*P* = 0.06).

## 10. Application versus Circulating Anti-IgE

All of these findings indicate that the CM when applied topically act on peripheral targets in the same fashion as do injections of the monoclonal humanized anti-IgE antibody omalizumab (Xolair, Genentech/Novartis) [12]. Both bind to the Fc portion of antibodies. The action of the CM, however, is immediate and probably acts on both mast cells seated IgE as well as IgE in the local circulation and interstitium, since the effects are abrupt, while the production and action of the monoclonal antibody takes place only over time and is said to be precluded from activity on IgE already seated on mast cells. Although we have no proof of this, it seems likely that CM attachment to local interstitial IgE, like Xolair, will down regulate the density of the IgE receptor on regional mast cells if applied over time. 

Since “application” of the CM easily provides the dose and concentration necessary (about 1/250 of common intravascular dosages) for inhibition of all local mast cell activating processes, it is significant to consider the various possible clinical applications which include the nose (allergic rhinitis), the eye (allergic conjunctivitis), the bronchi (asthma), the skin (atopic dermatitis), the esophagus (eosinophilic esophagitis), and possibly intra-articular spaces, the colon, and any other space open to installation or catheterization in which the mast cells are believed to play a significant role.

## 11. Technical Note

As an additional note, it must be realized that the potential of CM to inhibit competing antigens for attachment to antibodies makes it important to be sure that in the presence of CM in the circulation, the results of assays that depend on correct antigen-antibody dynamics, such as ELISA, are regarded with suspicion. This was first noted in a Japanese study, where tumor antigens were assayed and found to be less than expected quantitatively. It was noted by the authors that this occurred in several assays, where the patients had recently received intravascular CM and the association was made, but the explanation for the association was not forthcoming [5].

## 12. Conclusions

We are aware now that CM have potential pharmacologic applications beyond conventional opacification in X-ray studies. They are unique for small molecules in that they will inhibit ANY antigen-antibody reaction. Principles gleaned from research in CM physiology have suggested to us a new and completely unique approach to the treatment of hypersensitivity reactions. The abrupt inhibition of release of heparin and other mast cell products by sufficiently concentrated CM applied topically, and the profound inhibition of contact system activation is the key to this. 

While our research to date highlights CM inhibition of mast cells and symptoms of acute allergic rhinitis, we are aware that other cellular effectors including dendritic cells and monocytes, which are essential in the pathogenesis of chronic allergic rhinitis, atopic dermatitis, and asthma, also respond to upregulated IgE receptors that potentially may be inhibited in a similar fashion. In this regard, we have had encouraging results in treating a mouse model of atopic eczema, and expect similar results in humans. 

It is also possible that CM injected intravascularly in sufficient concentrations to bind to IgE in the circulation or on mast cells with the potential to compete with sensitizing antigens can be considered in the therapy of severe anaphylaxis. We have already demonstrated that this is possible in rats (Figure 5), and there is some evidence that the injection of epinephrine in these circumstances is not always accomplishing what is expected [13]. This approach, however, would entail considerable additional research, since suboptimal concentrations could activate mast cell anaphylaxis.
